# Weighted corrected covered area (wCCA): A measure of informational overlap among reviews

**DOI:** 10.1017/rsm.2025.19

**Published:** 2025-04-24

**Authors:** Xiangji Ying, Konstantinos I Bougioukas, Dawid Pieper, Evan Mayo-Wilson

**Affiliations:** 1 Department of Epidemiology, University of North Carolina Gillings School of Global Public Health, Chapel Hill, NC, USA; 2 Department of Hygiene, Social-Preventive Medicine & Medical Statistics, School of Medicine, Faculty of Health Sciences, Aristotle University of Thessaloniki, University Campus, Thessaloniki, Central Macedonia, Greece; 3 Center for Health Services Research, Brandenburg Medical School (Theodor Fontane), Rüdersdorf, Germany; 4 Faculty of Health Sciences Brandenburg, Brandenburg Medical School (Theodor Fontane), Institute for Health Services and Health System Research, Rüdersdorf, Germany

**Keywords:** weighted corrected covered area, corrected covered area, overlap, overview

## Abstract

When conducting overviews of reviews, investigators must measure and describe the extent to which included systematic reviews (SRs) contain the same primary studies. The corrected covered area (CCA) quantifies overlap by counting primary studies included across a set of SRs. In this article, we introduce a modification to the CCA, the weighted CCA (wCCA), which accounts for differences in information contributed by primary studies. The wCCA adjusts the original CCA by weighting studies based on the square roots of their sample sizes. By weighting primary studies according to their precision, wCCA provides a useful and complementary representation of overlap in evidence syntheses .

## Highlights

### What is already known

- Systematic reviews (SRs) commonly “overlap” by including the same primary studies.
- The corrected covered area (CCA) quantifies overlap by counting primary studies included across a set of SRs. It treats all primary studies equally.

### What is new

- When quantifying the amount of overlap between SRs, researchers should consider the amount of information that each study contributes.
- Study sample size is a useful indicator of the relative information that individual studies contribute to a SR; for example, it is related to the precision of estimates included in meta-analyses.
- We introduce the weighted CCA (wCCA), a quantitative measure of informational overlap across SRs. wCCA weights the primary studies using the square root of their sample size.

### Potential impact for RSM readers

- The wCCA is a novel tool for authors of overviews of SRs, which can provide a nuanced description of overlap among SRs in an overview.

## 1 Introduction

The rate of producing systematic reviews (SRs) continues to increase, with SRs exceeding randomized controlled trials (RCTs) in some fields.^1^ ^,^ ^2^ This surge has raised concerns about the potential for overlapping and redundant SRs.^2^ ^,^ ^3^

Overviews of SRs (also known as umbrella reviews, meta-reviews, or reviews of reviews) synthesize findings from multiple SRs that might include some of the same primary studies. Double-counting primary studies can magnify certain findings and skew overall conclusions. Overview authors often need to quantify overlap to inform their approaches to handling overlapping SRs and to describe the extent of overlap across the included SRs.^4^ Reporting guidelines for overviews of SRs recommend specifying the approaches used to illustrate or quantify overlap across SRs.^5^ ^–^ ^7^ However, methods to describe and to handle overlap are often reported poorly.^8^ ^,^ ^9^

Various methods have been devised to represent overlap in SRs, including tabular, graphical, and quantitative approaches.^10^ ^,^ ^11^ The corrected covered area (CCA)^12^ is a commonly used quantitative measure of the frequency with which primary studies are included in a set of SRs adjusted for the number of unique primary studies in the SRs.

Because the CCA method treats all included studies equally, it does not fully capture the extent of shared information across SRs. For example, SRs with many overlapping studies might have very different results if nonoverlapping studies are much larger than overlapping studies. Conversely, two SRs with few overlapping studies might have similar results if overlapping studies contribute most of the information in both SRs.

To quantify informational overlap, we propose a modification to the CCA, the weighted CCA (wCCA), which adjusts the original CCA by weighting primary studies according to their sample sizes.

## 2 Calculating wCCA

To compute the CCA, researchers first construct a citation matrix by listing primary studies in rows and listing SRs in columns.^12^ ^,^ ^13^ It is calculated using the formula CCA = (*N* – *r*) / ((*r* * *c*) – *r*), where *N* is the number of occurrences of primary studies across all SRs, *r* is the number of unique primary studies, and *c* is the number of SRs.^12^ ^,^ ^13^ The degree of study overlap might be interpreted as shown in Box 1.^12^ ^,^ ^13^

Box 1. Example of CCA thresholds and interpretation*

- 0% to 5%: ‘Slight study overlap’
- 6% to 10%: ‘Moderate study overlap’
- 11% to 15%: ‘High study overlap’
- >15%: ‘Very high study overlap’

*The thresholds serve as guidelines rather than strict rules.

To compute the wCCA, researchers incorporate the sample sizes of primary studies as follows:

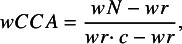


*where:*

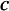
 *is the number of overlapping SRs (i.e., number of columns in the citation matrix).*

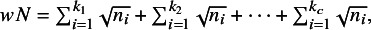
 *where* 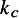 *represents the number of primary studies included in the c-th SR, and* 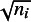 *is the square root of the sample size of each primary study. wN is the sum of the square roots of the sample sizes of all primary studies, aggregated across all SRs.*

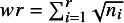
 *, where r is the number of unique primary studies (i.e., number of rows in the citation matrix). wr represents the sum of the square roots of sample sizes for unique primary studies, where each primary study is counted only once.*

The formula adapts the structure of CCA, replacing the sum of study counts with the sum of square roots of study sample sizes. This modification ensures that each primary study’s contribution is proportional to its square root of sample size. The square root is used instead of the raw sample size because it better aligns with the precision (e.g., standard error) of individual study estimates and their corresponding weights in meta-analyses (i.e., standard error is the standard deviation divided by the square root of the sample size). Using the square root function, smaller studies contribute to the index without being excessively overshadowed by larger studies. Thus, large studies are given greater weight than small studies—without being dominant—reflecting their typical influence on meta-analyses and SR conclusions.^14^

Like the CCA, the above formula can be modified to account for structural missingness,^15^ such as studies not included in an SR because of publication after the SR, as described in Appendix 1 of the Supplementary Material.

## 3 Considerations for using sample size as weight

Sample size is a useful proxy for the relative information that each study contributes to a SR. Although other metrics, such as the inverse of variances/standard errors of the effect sizes and the weights used in random-effects meta-analyses, could provide more precise estimates of informational overlap for individual outcomes, they are typically only available when meta-analyses are conducted. In contrast, the sample size of primary studies is commonly reported in SRs, regardless of whether a meta-analysis is conducted, as recommended in the Preferred Reporting Items for Systematic reviews and Meta-Analyses (PRISMA 2020) reporting guidelines.^16^ Moreover, using sample size enables wCCA to be calculated even when studies use different analytical methods. For instance, wCCA can be computed even if one primary study reports the intervention effect as a risk ratio and another uses risk difference. This flexibility makes wCCA a practical and accessible metric for a wide range of SR contexts.

**Figure 1:**
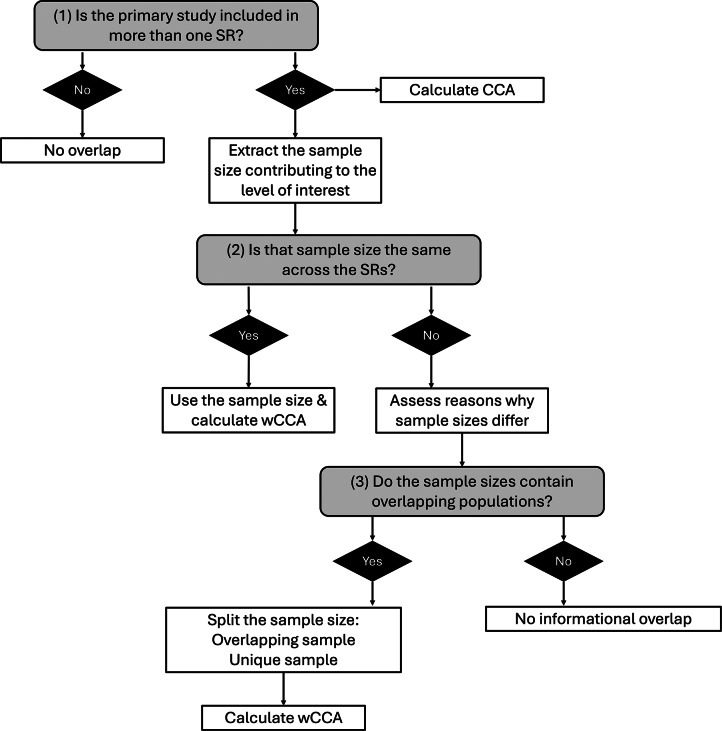
Flow chart to assess overlap.

SRs often contain multiple outcomes and analyses, and an overview might not focus on all of them. The sample size contributing to the level of interest (e.g., comparison, outcome, effect measure) should be used (Figure 1). For example, if a primary study includes three arms but the SR focuses on the comparison of two arms, the sample size of those two arms should be used rather than the total sample size. If multiple SRs report different sample sizes for the same primary study, it is important to investigate the reasons for the discrepancy. For example, differences might arise if one SR excludes participants who violated the protocol while another SR includes all randomized participants. Because wCCA uses the square root of the sample size, choosing between two similar numbers will be inconsequential in many cases. As a rule, the smaller of the sample sizes might be considered the overlapping population (i.e., the participants included in more than one SR’s results). Clear mapping of SRs to primary studies and the specific level of interest (e.g., outcomes, comparisons) is essential before calculating wCCA to ensure it accurately reflects the overlap of interest.

For cluster RCTs, the effective sample size (i.e., the sample size adjusted for the design effect) should be used, if available.

## 4 Illustrative examples calculating wCCA

Example 1: Overlap among SRs of RCTs.

**Figure 2:**
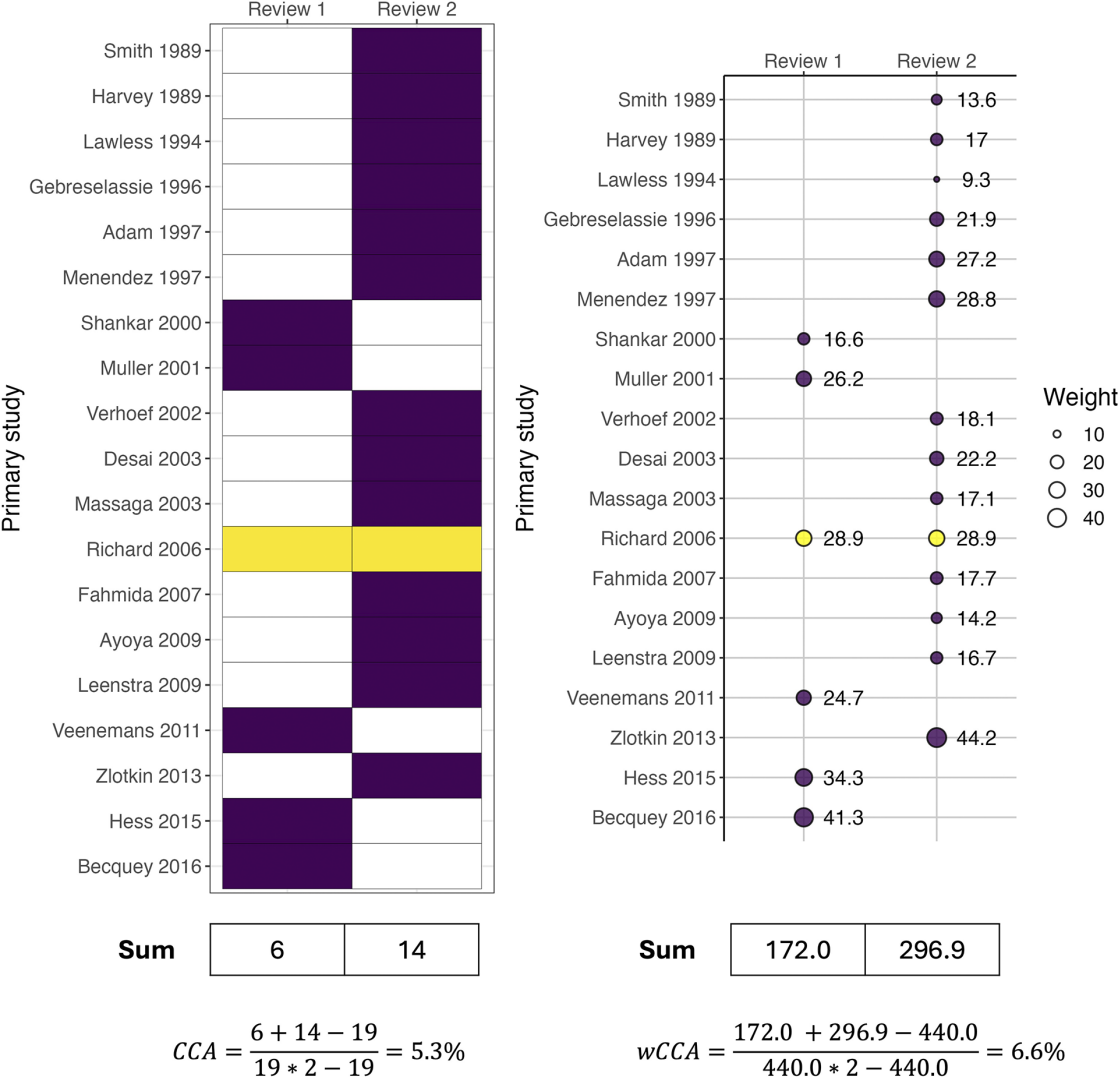
Illustration of CCA and wCCA calculations for overlap between SRs of RCTs (see Appendix 1 sTable 1 for tabular data; Appendix 3 for R code; and Appendix 4 for analysis-ready data).

Suppose researchers are conducting an overview of SRs on the effects of mineral supplements on malaria incidence in children. They decide to include one Cochrane review on zinc supplementation,^17^ which includes 6 trials assessing malaria incidence, and another Cochrane review of 14 trials on iron supplementation,^18^ along with other SRs. They find that the two SRs share one RCT in common. To quantify the overlap, they calculate CCA is 5.3%, indicating a slight overlap (Figure 2). They also decide to calculate a wCCA by extracting the sample sizes of the primary RCTs from the forest plots of interest in both SRs. The sample size of the shared RCT (*n* = 836) is the same in both SRs. Using the wCCA formula, they calculate wCCA is 6.6%, slightly higher than the CCA. If the sample size of the shared trial were 183 or 2,836, the wCCA would be 3.2% and 11.5%, respectively, showing greater deviation from the CCA. See Appendix 2 of the Supplementary Material for a worked example of quantifying overlap among more than two SRs.

Example 2: Overlap among SRs of observational studies.

Suppose researchers are conducting an overview of SRs that examine the association between allium vegetables and various cancers. They decide to include one SR that investigates the association of allium vegetables with upper aerodigestive tract cancers (nasal cavity, pharynx, larynx, oral cavity, and esophagus)^19^ and another SR that examines garlic consumption and its association with gastric cancer,^20^ among other SRs. When summarizing the association of garlic with cancers, they find that these two SRs share three primary studies that investigate both esophageal and gastric cancers.

**Figure 3:**
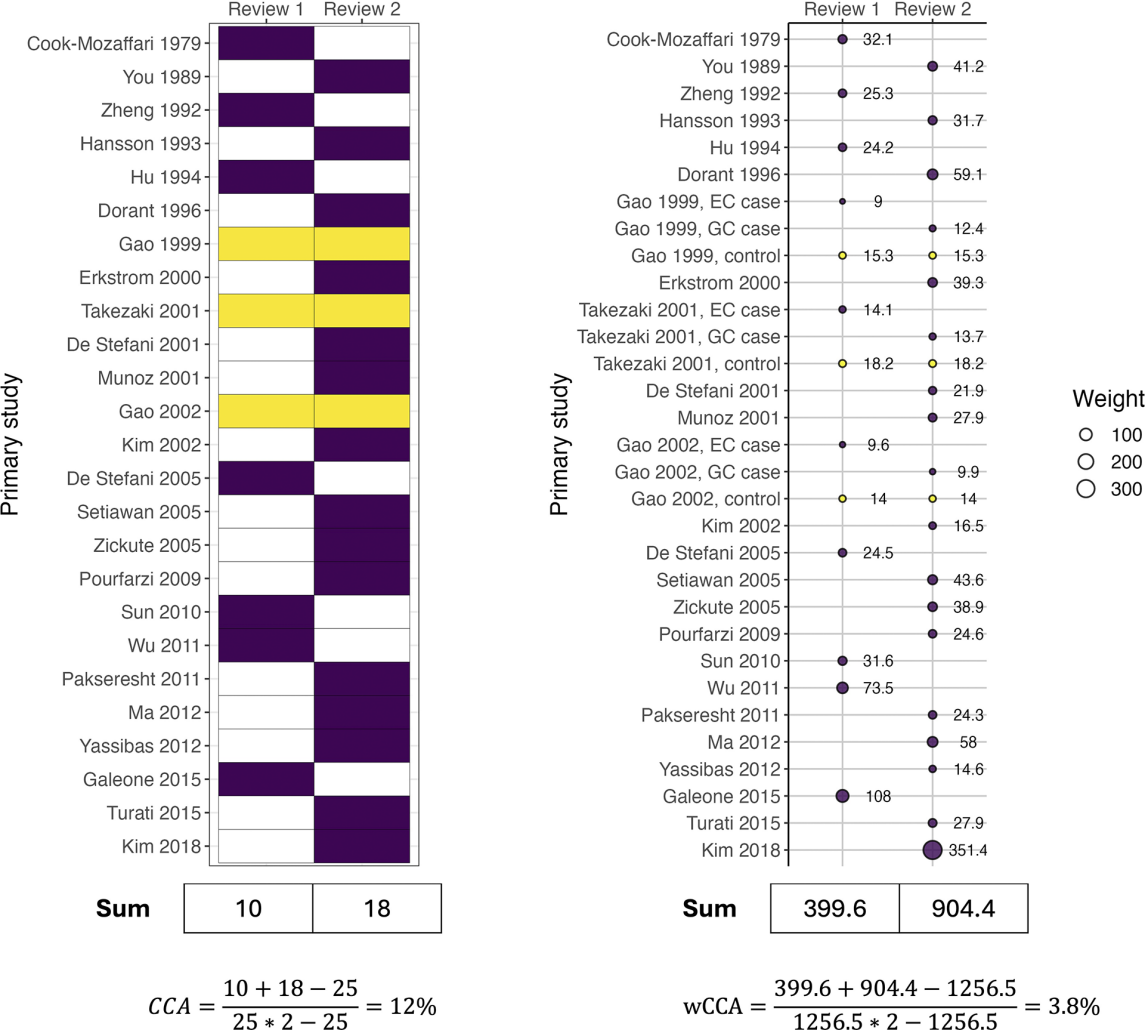
Illustration of CCA and wCCA calculations for overlap between SRs of observational studies (see Appendix 1 sTable 2 for tabular data; Appendix 3 for R code; and Appendix 4 for analysis-ready data).

They calculate the CCA is 12% (Figure 3), indicating a high overlap (Box 1). Next, they calculate the wCCA (Figure 3). Extracting the sample sizes of the primary studies from Table 1 of each SR, they observe that the three shared studies are case–control studies. Upon reviewing the original articles, they find the control group data are included in both SRs, but the cases differ (i.e., esophageal cancer cases contribute to one SR and gastric cancer cases to the other). Therefore, only the control group contributes overlapping information to the overview and the cases are unique in each SR. The researchers separate the overlapping and unique parts of the shared studies in the calculation, and they calculate a wCCA of 3.8%, considerably lower than the CCA.

## 5 Implications and interpretations of wCCA

The wCCA is a weighted version of the CCA. CCA and wCCA will produce similar results when each study in a set of SRs contributes a comparable amount of information. When study sizes vary, wCCA can offer additional information to the CCA alone.

The degree of overlap should be discussed in the context of the posed research questions and the topic’s scope (i.e., whether broad or narrow), as well as the data management decisions made during the study selection, data collection, and synthesis processes. When interpreting wCCA, researchers might apply the cutoff values used for CCA (Box 1). These relatively low cutoffs are chosen because SRs in an overview typically address different research questions within an overall topic. When researchers have attempted to minimize overlap by selecting the “best” SR for each sub-topic or by re-estimating results using primary study data, excessive overlap (e.g., two SRs overlap in 8 out of 10 studies) is relatively rare. As with CCA, these cutoffs serve as guidelines rather than rigid rules. Both CCA and wCCA are aggregate-level indices that describe the extent of overlap across a set of reviews. A low overall overlap does not necessarily indicate minimal duplicate information within all individual reviews.^21^ Therefore, decisions about whether to include a particular review in an overview should not be based solely on the CCA or wCCA value.

## 6 Limitations

Computing wCCA requires more information than CCA. Quantifying overlap using either wCCA or CCA might be challenging when dealing with dozens or even hundreds of SRs, especially if SRs include many studies. Other approaches for describing overlap and related concerns might be appropriate when SRs reporting quality is low. For example, neither wCCA nor CCA can be calculated when SRs do not describe or reference included primary studies.

## 7 Conclusions

The wCCA complements existing measures of overlap in overviews of SRs. It describes informational overlap across SRs using the square roots of sample sizes from primary studies. It enables a more nuanced assessment of overlap compared with CCA alone.

## Supporting information

Ying et al. supplementary material Ying et al. supplementary material

## Data Availability

R code for generating Figures 2 and 3 (including the calculations for CCA and wCCA in these examples) along with the R code for the worked example in Appendix 2 of the Supplementary Material can be found in Appendix 3 of the Supplementary Material.

## Supplementary material

To view supplementary material for this article, please visit http://doi.org/10.1017/rsm.2025.19.
