# Supplementary material for: Weighted corrected covered area (wCCA): A measure of informational overlap among reviews
Source: Res Synth Methods. 2025 Apr 24;16(4):701–8. doi: 10.1017/rsm.2025.19 (PMC12527530; doi:10.1017/rsm.2025.19)
Supplement: Ying et al. supplementary material [file S1759287925000195sup001.zip › Appendix 2_Final_20250410.docx]

Appendix 2. Worked example of weighted CCA calculations for more than two overlapping systematic reviews (see Appendix 3 for R code)

| **Study #** | **Study** | **Sample size (n)** | **Weight (**$\sqrt{\boldsymbol{n}}\boldsymbol{)}$ | | | |
| --- | --- | --- | --- | --- | --- | --- |
|  |  |  | **Unique** | **Review 1** | **Review 2** | **Review 3** |
| 1 | Smith 1989 | 186 | 13.6381817 |  | 13.6381817 |  |
| 2 | Harvey 1989 | 288 | 16.9705627 |  | 16.9705627 |  |
| 3 | Lawless 1994 | 86 | 9.2736185 |  | 9.2736185 |  |
| 4 | Gebreselassie 1996 | 480 | 21.9089023 |  | 21.9089023 |  |
| 5 | Adam 1997 | 738 | 27.1661554 |  | 27.1661554 |  |
| 6 | Menendez 1997 | 832 | 28.8444102 |  | 28.8444102 |  |
| 7 | Shankar 2000 | 274 | 16.5529454 | 16.5529454 |  |  |
| 8 | Muller 2001 | 685 | 26.1725047 | 26.1725047 |  |  |
| 9 | Verhoef 2002 | 328 | 18.1107703 |  | 18.1107703 |  |
| 10 | Desai 2003 | 491 | 22.1585198 |  | 22.1585198 |  |
| 11 | Massaga 2003 | 291 | 17.0587221 |  | 17.0587221 |  |
| 12 | Richard 2006 | 836 | 28.9136646 | 28.9136646 | 28.9136646 |  |
| 13 | Fahmida 2007 | 314 | 17.7200451 |  | 17.7200451 |  |
| 14 | Ayoya 2009 | 202 | 14.2126704 |  | 14.2126704 |  |
| 15 | Leenstra 2009 | 279 | 16.7032931 |  | 16.7032931 |  |
| 16 | Veenemans 2011 | 612 | 24.7386338 | 24.7386338 |  |  |
| 17 | Zlotkin 2013 | 1958 | 44.2492938 |  | 44.2492938 | 44.2492938 |
| 18 | Hess 2015 | 1178 | 34.3220046 | 34.3220046 |  |  |
| 19 | Becquey 2016 | 1705 | 41.2916456 | 41.2916456 |  |  |
| 20 | Jones 2019 | 100 | 10 |  |  | 10 |
| 21 | Doe 2022 | 400 | 20 |  |  | 20 |
| **Count of studies** | | | **21** | **6** | **14** | **3** |
| **Sum of weights** | | | **470.0065441** | **171.991399** | **296.92881** | **74.2492938** |

$$CCA=\frac{6+14+3-21}{21*3-21}=4.7\%$$

$$wCCA=\frac{171.99+296.93+74.25-470.01}{470.01*3-470.01}=7.8\%$$
