# Supplementary material for: Weighted corrected covered area (wCCA): A measure of informational overlap among reviews
Source: Res Synth Methods. 2025 Apr 24;16(4):701–8. doi: 10.1017/rsm.2025.19 (PMC12527530; doi:10.1017/rsm.2025.19)
Supplement: Ying et al. supplementary material [file S1759287925000195sup001.zip › Appendix 1_Final_20250410.docx]

Appendix 1. Formula of adjusted weighted corrected covered area

Bracchiglione and colleagues proposed an adaptation to the original CCA formula to account for structural missingness:^1^

$$adjusted CCA=\frac{N-r}{r\cdot c-r-X}$$

where X represents the count of structural missingness—studies that cannot be included in certain reviews, such as those published after a review. In this formula, the numerator N−r reflects the number of duplicate counts. X is subtracted only in the denominator, as structurally missing studies cannot contribute to duplicate counts. This adjustment results in a value larger than the original CCA, which accounts for the impact of structural missingness.

For the wCCA, we maintained the same structural logic but adapted it to incorporate weighted contributions:

$$adjusted wCCA=\frac{wN-wr}{wr\cdot c-wr-wX}$$

*Where:*

- $c$ *is the number of overlapping systematic reviews (i.e., number of columns in the citation matrix).*
- $wN=\sum_{i=1}^{k_{1}} \sqrt{n_{i}}+\sum_{i=1}^{k_{2}} \sqrt{n_{i}}+\ldots+\sum_{i=1}^{k_{c}} \sqrt{n_{i}},$*where* $k_{c}$ *represents the number of primary studies included in the c-th systematic review, and* $\sqrt{n_{i}}$ *is the square root of the sample size of each primary study. wN is the sum of the square roots of the sample sizes of all primary studies, aggregated across all systematic reviews.*
- $wr=\sum_{i=1}^{r} \sqrt{n_{i}}$*, where r is the number of unique primary studies (i.e., number of rows in the citation matrix). wr represents the sum of the square roots of sample sizes for unique primary studies, where each primary study is counted only once.*
- $wX=\sum_{i=1}^{x} \sqrt{n_{i}}$*, where x is the number of unique primary studies that form structural missingness. wX represents the sum of the square roots of sample sizes from structural missingness.*

Reference

1. Pérez-Bracchiglione J, Meza N, Bangdiwala SI, et al. Graphical Representation of Overlap for OVErviews: GROOVE tool. Res Synth Methods. 2022;13(3):381-388. doi:10.1002/jrsm.1557

sTable 1. Figure 2 Data

| **Study #** | **Study** | **Sample size (n)** | **Weight (**$\sqrt{\boldsymbol{n}}\boldsymbol{)}$ | | |
| --- | --- | --- | --- | --- | --- |
|  |  |  | **Unique** | **Review 1** | **Review 2** |
| 1 | Smith 1989 | 186 | 13.6381817 |  | 13.6381817 |
| 2 | Harvey 1989 | 288 | 16.9705627 |  | 16.9705627 |
| 3 | Lawless 1994 | 86 | 9.2736185 |  | 9.2736185 |
| 4 | Gebreselassie 1996 | 480 | 21.9089023 |  | 21.9089023 |
| 5 | Adam 1997 | 738 | 27.1661554 |  | 27.1661554 |
| 6 | Menendez 1997 | 832 | 28.8444102 |  | 28.8444102 |
| 7 | Shankar 2000 | 274 | 16.5529454 | 16.5529454 |  |
| 8 | Muller 2001 | 685 | 26.1725047 | 26.1725047 |  |
| 9 | Verhoef 2002 | 328 | 18.1107703 |  | 18.1107703 |
| 10 | Desai 2003 | 491 | 22.1585198 |  | 22.1585198 |
| 11 | Massaga 2003 | 291 | 17.0587221 |  | 17.0587221 |
| 12 | Richard 2006 | 836 | 28.9136646 | 28.9136646 | 28.9136646 |
| 13 | Fahmida 2007 | 314 | 17.7200451 |  | 17.7200451 |
| 14 | Ayoya 2009 | 202 | 14.2126704 |  | 14.2126704 |
| 15 | Leenstra 2009 | 279 | 16.7032931 |  | 16.7032931 |
| 16 | Veenemans 2011 | 612 | 24.7386338 | 24.7386338 |  |
| 17 | Zlotkin 2013 | 1958 | 44.2492938 |  | 44.2492938 |
| 18 | Hess 2015 | 1178 | 34.3220046 | 34.3220046 |  |
| 19 | Becquey 2016 | 1705 | 41.2916456 | 41.2916456 |  |
| **Count of studies** | | | **19** | **6** | **14** |
| **Sum of weights** | | | **440.0065441** | **171.991399** | **296.92881** |

$$CCA=\frac{6+14-19}{19*2-19}=5.3\%$$

$$wCCA=\frac{171.99+296.93-440.01}{440.01*2-440.01}=6.6\%$$

sTable 2. Figure 3 Data

| **Study #** | **Study** | **Sample size (n)** | **Weight** ($\sqrt{n})$ | | |
| --- | --- | --- | --- | --- | --- |
|  |  |  | **Unique** | **Review 1** | **Review 2** |
| 1 | Cook-Mozaffari 1979 | 1032 | 32.1247568 | 32.1247568 |  |
| 2 | You 1989 | 1695 | 41.1703777 |  | 41.1703777 |
| 3 | Zheng 1992 | 642 | 25.3377189 | 25.3377189 |  |
| 4 | Hansson 1993 | 1007 | 31.7332633 |  | 31.7332633 |
| 5 | Hu 1994 | 588 | 24.2487113 | 24.2487113 |  |
| 6 | Dorant 1996 | 3492 | 59.0931468 |  | 59.0931468 |
| 7 | Gao 1999, EC case | 81 | 9 | 9 |  |
| 7 | Gao 1999, GC case | 153 | 12.3693169 |  | 12.3693169 |
| 7 | Gao 1999, control | 234 | 15.2970585 | 15.29705854 | 15.2970585 |
| 8 | Erkstrom 2000 | 1547 | 39.3319209 |  | 39.3319209 |
| 9 | Takezaki 2001, EC case | 199 | 14.106736 | 14.106736 |  |
| 9 | Takezaki 2001, GC case | 187 | 13.6747943 |  | 13.6747943 |
| 9 | Takezaki 2001, control | 333 | 18.2482876 | 18.2482876 | 18.2482876 |
| 10 | De Stefani 2001 | 480 | 21.9089023 |  | 21.9089023 |
| 11 | Munoz 2001 | 777 | 27.8747197 |  | 27.8747197 |
| 12 | Gao 2002, EC case | 93 | 9.64365076 | 9.64365076 |  |
| 12 | Gao 2002, GC case | 98 | 9.89949494 |  | 9.89949494 |
| 12 | Gao 2002, control | 196 | 14 | 14 | 14 |
| 13 | Kim 2002 | 272 | 16.4924225 |  | 16.4924225 |
| 14 | De Stefani 2005 | 600 | 24.4948974 | 24.4948974 |  |
| 15 | Setiawan 2005 | 1902 | 43.611925 |  | 43.611925 |
| 16 | Zickute 2005 | 1516 | 38.9358447 |  | 38.9358447 |
| 17 | Pourfarzi 2009 | 604 | 24.5764115 |  | 24.5764115 |
| 18 | Sun 2010 | 1000 | 31.6227766 | 31.6227766 |  |
| 19 | Wu 2011 | 5399 | 73.4778878 | 73.4778878 |  |
| 20 | Pakseresht 2011 | 590 | 24.2899156 |  | 24.2899156 |
| 21 | Ma 2012 | 3365 | 58.00862 |  | 58.00862 |
| 22 | Yassibas 2012 | 212 | 14.5602198 |  | 14.5602198 |
| 23 | Galeone 2015 | 11672 | 108.037031 | 108.037031 |  |
| 24 | Turati 2015 | 777 | 27.8747197 |  | 27.8747197 |
| 25 | Kim 2018 | 123484 | 351.402903 |  | 351.402903 |
| **Count of studies** | | | **25** | **10** | **18** |
| **Sum of weights** | | | **1256.44843** | **399.639513** | **904.354265** |

$$CCA=\frac{10+18-25}{25*3-25}=12\%$$

$$wCCA=\frac{399.64+904.35-1256.45}{1256.45*2-1256.45}=3.8\%$$
